# The next phases of the Migrante Project: Study protocol to expand an observatory of migrant health on the Mexico—U.S. border

**DOI:** 10.3389/fpubh.2023.1032420

**Published:** 2023-04-17

**Authors:** Ana P. Martinez-Donate, Gudelia Rangel, Catalina Correa, Leah Bakely, Jesús Eduardo Gonzalez-Fagoaga, Ahmed Asadi González, Catalina Amuedo-Dorantes, Xiao Zhang, Carlos Magis-Rodriguez, Félice Lê-Scherban, Sylvia Guendelman, Emilio Parrado

**Affiliations:** ^1^Department of Community Health & Prevention, Dornsife School of Public Health, Drexel University, Philadelphia, PA, United States; ^2^Mexico Section of the U.S.-Mexico Border Health Commission, Tijuana, Baja California, Mexico; ^3^School of Medicine and Psychology, Autonomous University of Baja California (UABC), Tijuana, Baja California, Mexico; ^4^Department of Economics, University of California, Merced, Merced, CA, United States; ^5^School of Medicine, National Autonomous University of Mexico (UNAM), Mexico City, Mexico; ^6^Department of Pediatrics, University of Wisconsin, Madison, WI, United States; ^7^Department of Epidemiology & Biostatistics, Dornsife School of Public Health, Drexel University, Philadelphia, PA, United States; ^8^School of Social Welfare, University of California, Berkeley, Berkeley, CA, United States; ^9^Department of Sociology, University of Pennsylvania, Philadelphia, PA, United States

**Keywords:** Mexican migrants, migrant flows, health, healthcare access, Mexico-U.S. border

## Abstract

**Background:**

Mexican migrants traveling across the Mexico-United States (U.S.) border region represent a large, highly mobile, and socially vulnerable subset of Mexican nationals. Population-level health data for this group is hard to obtain given their geographic dispersion, mobility, and largely unauthorized status in the U.S. Over the last 14 years, the Migrante Project has implemented a unique migration framework and novel methodological approach to generate population-level estimates of disease burden and healthcare access for migrants traversing the Mexico-U.S. border. This paper describes the rationale and history of the Migrante Project and the protocol for the next phases of the project.

**Methods/design:**

In the next phases, two probability, face-to-face surveys of Mexican migrant flows will be conducted at key crossing points in Tijuana, Ciudad Juarez, and Matamoros (*N* = 1,200 each). Both survey waves will obtain data on demographics, migration history, health status, health care access, COVID-19 history, and from biometric tests. In addition, the first survey will focus on non-communicable disease (NCD), while the second will dive deeper into mental health and substance use. The project will also pilot test the feasibility of a longitudinal dimension with 90 survey respondents that will be re-interviewed by phone 6 months after completing the face-to-face baseline survey.

**Discussion:**

Interview and biometric data from the Migrante project will help to characterize health care access and health status and identify variations in NCD-related outcomes, mental health, and substance use across migration phases. The results will also set the basis for a future longitudinal extension of this migrant health observatory. Analyses of previous Migrante data, paired with data from these upcoming phases, can shed light on the impact of health care and immigration policies on migrants’ health and inform policy and programmatic responses to improve migrant health in sending, transit, and receiving communities.

## Background

International migration is one of the defining features of the 21st century and a recognized social determinant of health (1, 2). In 2021 alone there were 281 million international migrants worldwide (3). Globalization, economic inequalities, civil unrest, terrorism, and climate change are resulting in large population movements across the world with significant implications for the future of nations. In addition to the economic and societal impacts, migration, both authorized and unauthorized, has vast public health implications for migrants and for their sending, transit, and receiving communities. As the number of immigrants increases and as policies and social sentiments turn increasingly hostile towards immigrants, it is critical to establish research instruments to monitor the health and social conditions of mobile populations around the world. In this paper, we describe the rationale and history of the Migrante Project, a binational health observatory of migrant flows in the Mexico-U.S. border region, and present the objectives, design and methods of the next phases of this project. To the best of our knowledge, this study is unique in its focus on the health of mobile populations traveling through one of the most transited border regions in the world and, stands in contrast to other migrant health projects that emphasize static migrant populations in their sending or receiving communities.

### Migrante project’s rationale

Mexico has the second largest population living abroad, with about 11.2 million Mexicans living in the U.S. (4). This population is a highly mobile subset of Mexican nationals. Studies have documented extraordinary legal and economic conditions that increase social vulnerability and contribute to health disparities in this population (5, 6). Among Mexicans in the U.S., an estimated 48% are unauthorized immigrants, representing almost half of the total unauthorized immigrant population (4). Similarly, almost half of all Mexican immigrants live in poverty (7). Mexican immigrants also have the highest uninsured rate (53%) of any racial/ethnic group in the U.S. (7). Prevention, early detection, and adequate management are key factors for the control of infectious and non-communicable diseases, but limited access to healthcare poses a formidable obstacle for Mexican immigrants in the U.S. For example, one study found that among recent Mexican migrants, about half of those with diabetes and a third of those with hypertension were not aware of their condition (8). It is also estimated that as many as 1 in 4 Mexican migrants diagnosed with hypertension, hypercholesterolemia, and/or diabetes is not receiving any treatment for these conditions (6). In general, the foreign-born population in the U.S. gets diagnosed with HIV at later stages of the infection and enroll in care at much lower CD4+ counts than the U.S. born population (9). Data from previous research indicates that HIV testing levels are insufficient and rates of undiagnosed HIV infection are high among Mexican migrants (10, 11).

Conditions before, during, and after migration can affect the health of migrant populations. Some research suggests that the health of recently arrived Mexican migrants is better than that of their Mexican American counterparts, but deteriorates the longer they live in the U.S. (12, 13). A 2014 study showed that the health of recent Mexican migrants was about 60 percent more likely to have worsened within 1–2 years of migrating to the U.S. than the health of their counterparts who remained in Mexico (12). Limited access to prevention services, language barriers, cultural norms, separation from steady partners, and a lack of social support affect the sexual and reproductive care Mexican immigrants receive in the U.S. (14–18). Moreover, the obesogenic environment in many U.S. communities contributes to worsened diet quality and lowered physical activity levels among Mexican immigrants (19), and increases their risk of diabetes, obesity, hypertension, and heart disease (20, 21). Migration also deeply affects the emotional and psychological well-being of migrants. Isolation, separation from family, economic hardships, discrimination, and fear of deportation heighten the risk of developing mental health conditions and substance use issues among Mexicans in the U.S. (22, 23). However, studies have also found positive effects of migration on some health areas, such as smoking and use of preventive services (24, 25).

Changes in migrant sending and transit communities can affect the health profile of Mexican migrants to and from the U.S. during the pre-migration, transit, and return phases. Mexico is at the advanced stages of an epidemiologic transition, with increasing rates of obesity and non-communicable diseases (26, 27), such as diabetes and cardiovascular disease. New generations of migrants have been exposed to drug-related violence, both in their communities of origin and during their migration journey, at unprecedented rates (28). On the other hand, expansions of the Mexican health system have increased rates of health insurance among return migrants and non-migrants in Mexico (29).

Important changes in the volume, circularity, and demographics of Mexican migration have been observed in the last decade (30, 31). Most notably, the last years have witnessed the mass deportation of Mexican immigrants with established residence in and ties to the U.S. (32, 33). From 2009 to 2019, 1.5 million Mexican immigrants were deported (32). The point of entry into the U.S., traditionally concentrated in the western border region, has, since 2008, moved to central and eastern regions of the border (34). New Mexican communities are emerging in southern and central U.S. states led by migrants from non-traditional sending communities in rural central Mexico (35). Concurrently, socio-political factors have resulted in a large influx of migrants, including families and unaccompanied minors, from the Northern Triangle countries (El Salvador, Honduras, and Guatemala) seeking political asylum in the U.S. The Trump administration responded with the ‘Remain in Mexico’ policy, which required these asylum seekers to wait in Mexico for months, or even years, while their asylum petitions were processed (35). This policy might remain in effect under the Biden administration. More recently, with the COVID-19 pandemic, the U.S. government invoked Title 42, a rarely used U.S. public health law that allows for the summary expulsion of migrants without allowing them the opportunity to seek protection in the U.S. (36). This law, which as of January 2023 is still in place, has effectively closed the door to asylum seekers and resulted in a sizable population of mostly Central Americans stranded on the Mexican border, unable to reach their destination in the U.S. or go back to the violent and impoverished communities they fled. Forced displacements due to deportation or summary expulsion, and changes in the geography and profile of Mexican migration, can have important consequences for the health of immigrants and residents of their receiving, transit, and sending communities. For example, the unprecedented number of migrants returned to the Mexican border region over the last decade can link infectious disease risk to geographically distant communities in Mexico and the U.S. (37).

The large volume and vulnerability of the migrant flows traversing the Mexico-U.S. border region, and the important public health impacts of these flows on both the U.S. and Mexico, make it imperative to have instruments in place to monitor the health status, access to services, and health determinants of these mobile populations. While national and state-level surveys in the U.S. and Mexico can produce a portrait of the stationary Mexican immigrant and returned migrant populations, respectively, these instruments fail to capture the characteristics and changes in populations constantly moving North and South between sending and receiving communities, and through transit border regions. Over 350 million people cross the U.S.-Mexico border every year, a population made up of both lawful and unlawful migrants traveling by bus and train, in personal vehicles, rail and shipping containers and, on foot, among other means of transportation (38). Responding to the growing importance of the health and healthcare needs of people moving across international borders, the Migrante Project seeks to examine health conditions and access to healthcare among mobile populations traveling across the Mexico-U.S. border, one of the most traversed international border regions in the world. Migrante aims to provide an up-to-date perspective on the health of border crossers, including deported migrants, an understudied and vulnerable migrant population.

### Migrante Project history

The Migrante Project is a binational effort funded by the Eunice Kennedy Shriver National Institute of Child Health and Human Development (NICHD) and led by investigators at Drexel University (Philadelphia, PA) and the Mexico Section of the U.S.-Mexico Border Health Commission (henceforth USMBHC), in Tijuana (Mexico), with collaborators at other institutions in both the U.S. and Mexico. From 2007 to 2015, the Migrante Project focused on HIV infection, HIV-related risk behaviors, and healthcare access among Mexican migrant flows traveling to or deported through Tijuana, Mexico (16). In 2020, the Migrante Project was expanded from Tijuana to Matamoros and Ciudad Juárez to produce updated data on sexual and reproductive health outcomes from Mexican migrant flows crossing through other regions of the Mexican border. Cross-sectional survey data collected from 2009 through 2021 have substantially contributed to estimating prevalence levels, and factors associated with HIV risk (10, 16, 39, 40), substance use (41), health care service utilization (42–44), and COVID-19^46^ among Mexican migrants at various migration phases: Northbound migrants with and without prior international migration history, migrants traveling South after time spent in the U.S. or the Mexican border region, and migrants deported to the city of Tijuana. The findings have led to the establishment of free prevention clinics in five deportation stations along the Mexican border with funding from the Mexican Secretariat of Health.

The overall goal of the Migrante Project has been to collect critical migrant health data to identify health trends, healthcare needs, and risk and protective factors among Mexican and Central American migrants traveling through one of the busiest border regions in the world and to inform policies and programs aimed at protecting the health of this population.

In the next phases, the Migrante Project will conduct two new probability survey waves of key migrant flows (*N* = 1,200 each) traveling to the Mexico-U.S. border region. In the following sections, we describe the theoretical foundation, design, and measures of these next surveys and the potential of the Migrante observatory to serve as an international reference and model for the collection of migrant health data in other border regions.

### Theoretical framework

**Figure 1:**
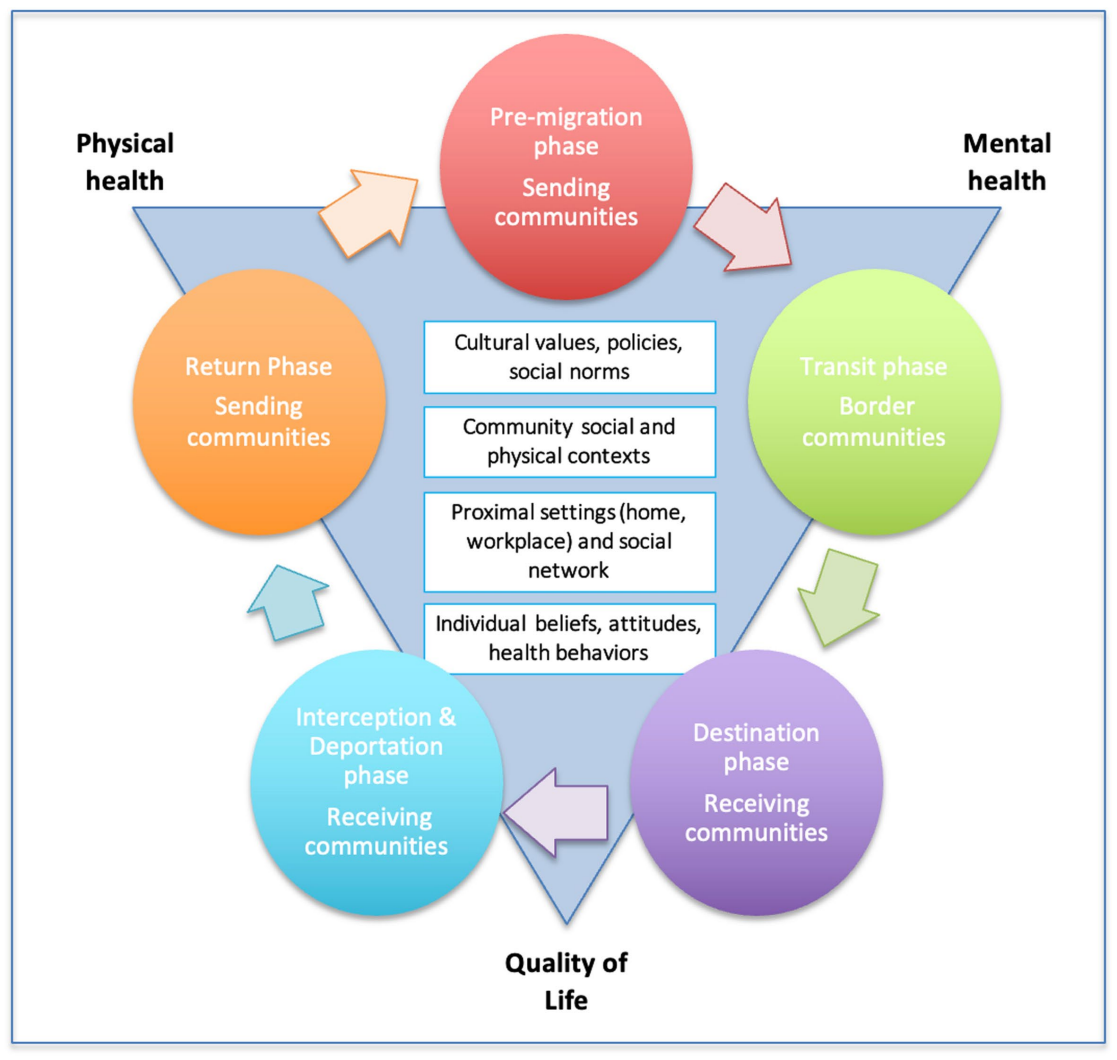
Theoretical framework of the Migrante Project.

The Migrante Project’s conceptual framework integrates elements from three complementary models. First, it incorporates *Zimmerman’s Migration Framework* (45), which defines migration as a process that involves a series of phases, each characterized by unique health risks and intervention opportunities. Five specific migration phases are distinguished by this framework: *pre-departure, transit, destination, interception, and return* (Figure 1). The framework highlights the importance of identifying and responding to the needs of migrant populations at all phases of migration. Second, this project is informed by the *Social Ecological Model* (46), which contends that health outcomes and behaviors are the result of ongoing and complex reciprocal interactions between individuals and the persons, objects, and symbols in their environment. Health and well-being are determined by a hierarchy of individual characteristics, proximal social networks, community-level factors (46), and the broader physical, social, legal, and cultural environment (47). Different constellations of factors influence migrant health across migration phases. Finally, *Andersen’s Model of Health Services Use In Immigrant Populations* (48) guides the Migrante Project’s examination of general and migration-specific determinants of health care utilization. *Predisposing* factors affect migrants’ perception of the need for services and their ability to cope with health problems. *Need* factors motivate migrants to obtain care. *Enabling* factors are individual and environmental factors that enable or impede migrants’ health care access and are amendable through policies. Macrostructural/contextual factors are social structures and community factors that constrain options for migrant populations ( 48).

## Methods/design

### Target population and recruitment goals

Each of the two new Migrante Project surveys will include 1,200 migrants at various transit points located in the Mexican border cities of Tijuana, Matamoros, and Ciudad Juárez. These three border cities were chosen strategically to produce the maximum scientific yield. Close to half of the U.S.-Mexico return migrant flow travels through them. By the end of June 2021, the percentage of deportation events from the U.S. going through these cities was 38% (49). Located in the west, central, and east border regions, these cities receive flows coming from and heading to a variety of sending and receiving regions in the U.S., Mexico, and other Latin American countries (30, 34–51). Moreover, they receive the flows of ‘returnee’ migrants from Central America that are either sent back to Mexico to wait for their refugee application under Title 42 or simply expelled from the United States.

**Figure 2:**
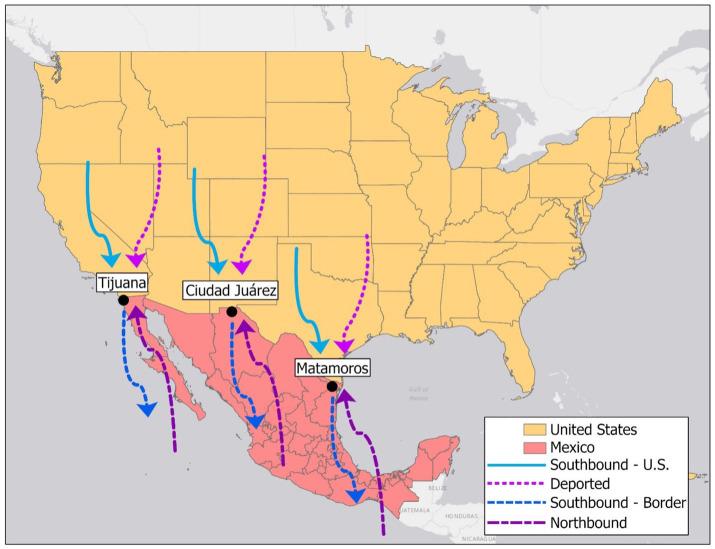
Migrant flows converging on the Mexico-U.S. border region and sampled by the Migrante Project.

Three key migrant flows will be sampled for each of these surveys in each city: Deported migrants (*N* = 300), Northbound migrants (*N* = 300), and Southbound migrants (*N* = 600). The latter flow will be further stratified into migrants traveling from the U.S. (*N* = 300) and migrants traveling South from the Mexican border region (*N* = 300). These migrants represent different migration phases and spatial trajectories (See Figure 2).

The *Deported Flow* is made up of migrants released at the Mexican border after being deported from the U.S., a growing and highly vulnerable migrant population. Data on health status and health care among this flow will provide information related to migrants at the *interception/deportation* phase.

The *Northbound Flow* is composed of migrants traveling North to the border region from their communities of origin in Mexico and/or Central America. This flow includes both individuals without (new migrants) or with (returnees) a previous migration history who are (re)engaging in internal or international migration for the first or subsequent times. Their health status is influenced by their migration history and recent exposure to the conditions present in their communities of origin. Data from migrants in this flow will represent the *pre-departure* and *return* phases.

The *Southbound Flow* comprises migrants returning to their sending communities after a stay in the U.S. or the Mexican border region. For those returning from the U.S., which include both more established immigrants as well as sojourners, the surveys will offer information on the health of migrants during the *international destination* phase. For migrants with a recent stay in the Mexican border region, the surveys will offer a snapshot of the health of migrants recently exposed to a high-risk *intermediate context/transit phase*, especially for HIV and substance use risk behaviors (37, 39). The focus on migrant flows vs. migrant stocks and the use of consistent metrics for the different phases will help us compare health indicators and identify changes in health and health determinants across migration stages and contexts and over time.

### Sampling methods

The design of the next two Migrante Project surveys is modeled after the *Surveys on Migration on the Northern and Southern Mexican Borders* [Encuesta sobre Migración en las Fronteras Norte y Sur (EMIF Norte and EMIF Sur), per their Spanish names and acronyms] (30). The EMIFs are two long-standing surveys (EMIF Norte started in 1993 and EMIF Sur dates back to 2004) conducted by *El Colegio de la Frontera Norte (COLEF)* and funded by various secretariats of the Mexican Government to assess and characterize the demographic and socio-economic profile of migrant flows crossing Mexico’s Northern and Southern borders. The EMIF methods rely on the premises that migrants represent mobile units that can be intercepted at certain times/places and that the majority of migrants traveling across the Mexico-U.S. and the Mexico-Guatemala borders do so by ground and through a handful of Mexican border cities that connect the border regions with the rest of Mexico and the U.S. or Guatemala (52, 53). Even migrants who cross the border through more remote border areas must first access the border region using the transportation infrastructure present in these cities. These dynamics create specific venues (e.g., airports, bus stations, migration facilities) and specific points (e.g., gates, doorways, through which migrants must cross when arriving or departing the Mexican border region) at which migrant flows narrow and where they can be effectively sampled. The Migrante Project’s methods involve observation, enumeration, and screening at these specific points to obtain probability-based samples of key migrant flows (52, 53).

As in previous Migrante Project phases, sampling for the next two surveys will use a two-stage sampling design with two dimensions (i.e., time and space) and two sampling stages for each study site. The geographic (space) dimension includes the sampling site and point within the site (if more than one). In Tijuana, Ciudad Juarez and Matamoros, the sampling sites will include the airport, the central bus station and the deportation station. The sampling points will be the doorway(s) to the passport control points and the gate(s) to the luggage claim area(s) in the airports; the ticket desks and the luggage claim areas in the bus stations; and the hallway area through which deportees exit the deportation stations after being cleared for departure. The time dimension consists of the day of the month and the survey shift (Night: 00:00–8:00; Morning/Afternoon: 8:00–16:00, and Evening: 16:00–24:00 h).

During the implementation of each survey wave, a stratified random sample of “space – time” pairs will be selected monthly for each city to determine the data collection schedule for each month. A rigorous process will be conducted independently for each city and migrant flow. First, for the geographic dimension, the selection process will proceed from sampling site to sampling point. Next, for the temporal dimension, the selection will proceed from day of the week to survey shift. With some exceptions, the probability of selection of units at each stage will be proportional to the volume of migrant flow the temporal and geographic units account for, based on our sampling frame. The sampling frame was generated in November of 2021 through continuous enumeration and screening during a 24/7 period.

### Screening and recruitment

During each scheduled survey shift, potential participants will be consecutively approached by a trained project staff member and screened for eligibility, while a second research assistant will enumerate every adult crossing by the sampling point. Field personnel will be Mexican natives hired by the USMBHC. Eligible individuals for the study will be 18 years or older, born in Mexico or other Latin American countries, fluent in Spanish, not residents of the study cities (except for deported migrants), travelling for labor reasons or change of residence or without a specific return date, and with no previous study participation in the Migrante Project survey wave for which they are being screened. Our measures will be administered exclusively in Spanish. Persons who are unable to answer questions due to mental or physical limitations will also be excluded from the study. Eligible individuals will be provided with study information, consented to participate in the study, and led to a quiet, private area within the survey site where the survey can be administered. Enumeration will continue until administration of the survey measures for a participant is completed. After completing the measures with one participant, the screening, recruitment, and enumeration process will be repeated through the end of the survey shift. Participants will be assigned a unique numeric code that will be used to link interview data and biological tests/samples. No names or other identifying information will be collected from participants.

### Measures

The next two Migrante Project survey waves consist of an interviewer-administered questionnaire and several biometric tests. The design of survey measures will be informed by our theoretical framework. The questionnaire will include general sections common to previous survey waves and new wave-specific sections (Table 1). As in previous waves, questionnaires collect information on demographics, socio-economic characteristics, health outcomes, and healthcare access, and socio-ecological variables. A detailed migration history will also be collected, with attention to places and dates of residence (states, counties, cities) since the time participants first migrated, as well as socioeconomic conditions, availability of social support, acculturation and acculturative stress (54). These factors are conceived of as individual and environmental determinants of health and health care access outcomes. In addition, the first of the next two survey waves (*N* = 1,200) will have sections focused on indicators of, and risk factors for, non-communicable diseases (i.e., NCD survey wave); and the second of the next two survey waves (*N* = 1,200) will explore the prevalence, risk factors and determinants of mental health and substance use (i.e., MHSU survey wave. Importantly, and as in the most recent Migrante survey, the new questionnaires will include COVID-19 related questions pertaining to infection, testing, treatment, and vaccination history; economic impacts of the pandemic; and adherence to prevention measures (e.g., social distancing, mask wearing, hand washing, etc.).

**Table 1:** Migrante Project survey measures and components (2022–2024).

Questionnaire		
Survey wave	Questionnaire section	Topics covered
2022–2023	Demographics and migration history	Demographics
2023–2024		Geographic information and migration history
		Detention and deportation history
		Migration plans
2022–2023 2023–2024	General Socio-ecological Health Determinants	Socio-economic conditions
		Social support
		Acculturation
		Acculturative stress
		Adverse Childhood Exposures
		Major lifetime stressors
2022–2023	Health Care Access and Utilization	Physical and mental health status and health limitations
2023–2024		Self-reported disease and chronic conditions
		Access to health care
		Alcohol and substance use
		Experiences during the last time they received health care
		Health-seeking border crossing practices
		Health insurance status
2022–2023 2023–2024	COVID-19 Measures	COVID-19 infection history, precautions, testing & vaccination, care seeking practices
2022–2023	Non-communicable Diseases (NCD)	Self-reported chronic conditions/morbidity
		Access to chronic disease prevention services
		Physical activity
		Diet
		Access to healthy foods at the community level
		Food Insecurity
		Perceived built and social environment
		Tobacco use
2023–2024	Mental Health & Substance Use	Mental health conditions
		Lifetime use of illicit and prescribed substances
		Age and migration context at first use
		Last 30-days substance use
		Last 12-months needle injection practices
Biological testing		
2021–2022	Biomarkers of Non-Communicable Disease	Glycated hemoglobin, lipid profile, blood pressure, weight, height, waist circumference, body fat
2022–2023	Biomarker of Stress	Salivary cortisol concentration levels

Survey questions for each migrant flow will be adapted to collect information about conditions, behaviors, and environmental factors experienced while migrants are in the migration context applicable to their migration phase: (a) sending communities in Mexico for Northbound migrants at the pre-migration and return phases; (b) the Mexican border region for Southbound migrants traveling from the border region; (c) receiving communities in the U.S. for migrants returning from the U.S.; and (d) receiving communities in the U.S. and detention settings for deported migrants. Most questions will use a 12-month timeframe, but this timeframe will be reduced according to the amount of time respondents were in their applicable migration context (e.g., sending communities, receiving communities, etc.), which will be determined early in the survey. Questionnaires will be pilot tested (*N* = 30) and revised as necessary to ensure comprehension by study participants.

The design of the measures will rely on instruments used in the Migrante Project’s previous phases (16, 44) and national or international studies that have included Mexican immigrants or Mexican nationals. A sample of previous questionnaires is available on our website^1^. The use of previous measures will allow us to make comparisons to determine time trends, as well as disparities in health status, risk, and access to treatment among Mexican migrants.

### Biometric tests

Participants in the NCD survey wave will have their weight, height, and waist circumference measured by trained program staff. They will also receive a lipid profile test that will include total cholesterol, high density lipids, triglycerides, low density lipids, and non-HDL using the portable analyzer CardioCheck™ (Polymer Technology Systems Diagnostics [PTS], Inc., Indianapolis, IN), a finger stick rapid test (55). Respondents will also be tested for glycated hemoglobin, a biomarker for diabetes that measures glycohemoglobin (HbA1c), which reflects the average blood glucose concentration over the course of the RBC lifespan. This test will be done with the A1CNow + portable analyzer (PTS Inc., Indianapolis, IN) (56), which also uses a finger stick whole blood rapid test. Respondents’ blood pressure will also be measured using a portable digital blood pressure monitor (Welch Allyn’s Connex® ProBP™ 3,400) (57) to assess systolic and diastolic blood pressure levels. Finally, skinfold thickness (in millimeters) will be measured using skin fold calipers to assess body fat composition. Measures will be taken on the mid-line of the back surface of the arm over the triceps muscle and on the inside surface of the calf and averaged for analyses.

As part of the MHSU survey wave, participants will be tested for cortisol levels using a saliva sample collected using a the Salimetrics Salivary Cortisol ELISA Kit (58) and SalivaBio Oral Swabs (59). Samples will be refrigerated, stored, frozen and shipped monthly to a Salimetrics concerted lab for analyses. Cortisol is considered a stress-responsive biomarker, linked to many physiological processes and health outcomes (60). Salivary concentration levels of cortisol are frequently used as markers of stress exposure (61).

### Data collection

Survey measures will be administered by local project staff trained to obtain informed consent, conduct structured interviews on sensitive topics, and administer biometric tests. Questionnaires and tests will be conducted in a private office (when available) or a quiet area within each sampling point. Screens will be used to increase privacy and participants’ comfort. Data collection will be conducted using the Qualtrics® offline survey software on iPads®. Results from all biometric measures (minus cortisol) will be entered into the protocols immediately after being obtained. In the case of cortisol levels, the results will be submitted to the program manager at Drexel University by the concerted lab. The program manager will use the unique participant ID to add the results to the datasets. Survey data collected in the field will be uploaded to the Qualtrics server at the end of each survey shift and downloaded and reviewed by the program manager at Drexel University on a weekly basis. These procedures will decrease cost and data entry errors and allow closer monitoring of data collection.

### Pilot longitudinal study component

The Migrante Project’s protocol also calls for recruiting ninety (90) participants into a longitudinal pilot component that will examine changes in health status, risks, and migration determinants of health over a 6-month period. These participants will be recruited from the NCD survey wave (30 from each migrant flow) and only in the city of Tijuana, for piloting purposes. Survey participants will be consecutively invited to participate in the six-month longitudinal pilot study until the target sample size of 90 has been reached. Participants who agree to participate will be given a pre-paid phone with $50 credit and a charger and asked to provide their contact information and the numbers of several contacts in order to be able to complete phone check-ins and a final 6-month follow-up interview. This phone follow-up interview will include all self-reported health and healthcare measures administered during the baseline interview as well as detailed migration information and socio-ecological variables for the 6-months between baseline and follow up.

To reduce attrition, research staff will attempt to contact participants *via* phone once a month and use voice and text message reminders over a one-week period, as necessary. If attempts to reach a participant *via* the provided phone are not successful, research staff will attempt to contact them using the phone number(s) of the contacts provided. If research staff is unable to establish contact within 14 days of the initial attempt, telephone service will be canceled. For each successful monthly check-in, participants will receive an additional $10 in phone credit or e-gift card. Pilot participants will receive an additional $50 in phone credit or e-gift card for completing a final 6-month follow-up interview.

The longitudinal component of the study is not designed to test for significant changes in health outcomes or to produce generalizable estimates. Rather, the aim of this longitudinal component is to test the suitability of the study methods for a future panel survey following participants over time. The main outcome of this component will be the recruitment, retention, and characteristics of this pilot sample.

### Training of personnel

All Migrante Project personnel participating infield activities and in contact with participants have received training on both research ethics and survey implementation from investigators at Drexel University and USMBHC, with support from *ad hoc* clinicians with expertise in biometric testing. All field personnel have been recruited from local communities by the Mexico Section of the USMBHC. The training comprises multiple sessions covering (a) screening, sampling, recruitment, and obtaining verbal consent from potential study participants; (b) administration of surveys, collection of specimen samples, and completion of biometric tests; (c) provision of counseling to participants who test positive for any of the biometric tests and their referral to further diagnostic services; (d) incentive disbursement, crisis management and practical ethics; (e) safe uploading of survey data to the server and troubleshooting of any issues arising with the iPads and the Qualtrics app; (f) biosafety protocols for risks related to COVID-19 and the collection and handling of biological specimens; and (g) ethics in human subjects research. The training includes a theoretical and practical component. Any new personnel will complete these trainings prior to engaging in any human subjects activities.

The survey procedures involve obtaining informed verbal consent from study participants. Trained research assistants will provide a thorough explanation of the survey, investigators, purpose of the study, procedures, risks and benefits. The consent process stresses that prospective participants have the right to refuse participation, decline to answer any questions, and stop at any time. While there are not direct benefits to study participants, we expect based on previous experience that some respondents will find it useful to learn about the results of the rapid tests applied as part of the surveys, such as cholesterol, glucose, high blood pressure, and height and weight. The results of these tests will be provided immediately to study participants, both verbally and in writing. Research staff will emphasize that these tests are not for diagnostic purposes and will advise participants on the need to check with a health provider to confirm any potentially abnormal findings. The field personnel will have a list of community health centers in the survey cities. Respondents will also be provided with the phone number of the Border Health Commission and will be encouraged to call if they need assistance finding out a community clinic in their place of destination.

As in previous Migrante phases, all field activities will be monitored and supervised by a local supervisor, who will report to the survey coordinator at the USMBHC and an overall project manager at Drexel University, who in turn will report weekly to the Site-Principal Investigator in Mexico and the Principal Investigator at Drexel. WhatsApp will be used to ensure ongoing communication within and across teams in Mexico and the U.S. All field personnel will receive detailed biweekly feedback on their performance to ensure data quality and rigor. Local supervisors will provide feedback to the investigators regarding all field operations and adjustments to the protocol will be made accordingly. Investigators, program managers, and local supervisors will continue to meet biweekly to review progress with data collection and troubleshoot any problems with survey implementation.

### Data analysis

Survey weights will be computed for each observation to obtain flow-level parameter estimates. Our weighting procedures will be modeled after those used during our previous Migrante Project phases (16, 43). The formula takes into account the complex survey design, the number of people who cross by the site, the number of people screened who did not qualify, and the number who refused to participate (49, 60). To estimate the levels of health care access and prevalence of health outcomes and health determinants among Mexican migrants traveling across the US-Mexico border, we will estimate weighted descriptive statistics (i.e., frequencies, percentages, means and standard deviations). Analyses will be conducted separately for each migration wave and flow, but data can be combined across waves and flows, depending on the outcome and the purpose of the analysis.

Furthermore, to calculate the association between migration phase and health care access, health outcomes, and theoretical health determinants, we will use staged logistic regression models, ordinary least squares (OLS) regression models, and multinomial regression models depending on the outcome examined. In stage 1, selected indicators of healthcare access will be regressed on migration phase (our main predictor), using the premigration phase (i.e., Northbound migrants without previous migration experience) as the reference. In next stages, predisposing (e.g., demographic, socio-economic, acculturation, psychosocial factors), need (e.g., health status, limitations), enabling (e.g., health insurance, transportation, incarceration, language barriers), and macrostructural/contextual factors (e.g., immigration status, reasons to migrate, state of destination) will be added to the model. Similarly, to examine the association between migration phase and NCD, mental health outcomes, and health behaviors, we will enter migration phase first, and subsequently introduce individual, inter-personal, and contextual variables.

Additionally, to explore the impact of health care and immigration policies on access to health care and the health status of returning Mexican migrants, we intend to use the survey data to perform some exploratory policy analyses. We will examine variations in health care access using data from the new surveys and data from previous Migrante Project surveys dating back to 2009. We will also explore the impact of selected federal, state, and local immigration enforcement initiatives on the well-being of this population. Most policy analyses will adopt a *difference-in-difference* approach to gage changes in our outcomes of interest (e.g., health outcome) on *treated* vs. *control* individuals *before* and *after* treatment (policy), replicating methods used in our previous work (62, 63). We will build a number of indices capturing the intensity of immigration enforcement at the county or the state level. The indices will take into account a variety of immigration enforcement initiatives. Our analyses also include checks for identification, heterogeneity, and robustness.

Finally, to test the feasibility of the longitudinal component, we will focus on descriptive analyses and simple bivariate analyses of response rates, contact and retention/attrition rates, and baseline differences between participants retained in the study and lost to follow up.

### Statistical power

The sample sizes were calculated using a publicly available sample size calculator^2^ to be able to produce reasonably narrow confidence intervals for our prevalence estimates of the most important study outcomes within the constraints of the funding resources available. Based on available prevalence rates for Mexican migrants or adults in Mexico for many of the outcomes of interest, power analyses indicate that our sample sizes will be sufficient to produce precise prevalence estimates of the most relevant NCD, mental health, substance use and healthcare outcomes (margin of error ranging 1.7% to 6.2%). These analyses also suggest enough power (> = 85%) to detect relatively small and moderate associations between migration phase and health indicators (odds ratios equal or greater than 1.35). We have estimated that we will also have adequate power levels (> = 85%) to explore moderate effects of immigration and health care policies on health and healthcare access (odds ratios equal or greater than 1.6).

### Timeline and preliminary descriptive statistics

Each of the next two Migrante Project survey waves is expected to be completed within a period of approximately 12 months. The most recent Migrante survey was completed between August 2020 and October 2021 with a total of 1,260 participants (~105% of the target sample size). By migration flow, cooperation rates (percent of participants who consented among those eligible) ranged from 13% to 52% for Northbound migrants; 28%–75% for migrants traveling South from the U.S.; 37%–70% for migrants traveling South from the Mexican border; and 72.5%–98% for migrants deported from the U.S. in the three survey cities for participants in the first survey wave. We expect similar response rates for the next two surveys.

## Discussion

Recent and potential public health threats and changes in health care policy, immigration practices, labor shortages, and social sentiments towards immigrants can have a profound impact on the make-up and health status of and health care access for migrants in the U.S. By the end of the current funding cycle, data will have been collected for the Migrante Project for over a decade (2009–2024). The continued data collection by this binational “migrant health observatory” will be critical to understanding the health and healthcare needs of migrants traveling through one of the world’s largest migration corridors (64). The data accrued to date have contributed to estimating HIV rates (10, 16) and related behaviors (41) among Mexican migrant flows traveling through the U.S.-Mexico border region. They have also increased our understanding of factors associated with migrants’ risk for HIV and other STIs, including contextual influences, acculturative stress, and migration experiences. In addition, these data have produced knowledge about health care access (42–44) among these disenfranchised populations, including access to preventive screenings (65) and vaccinations (66) and the potential to increase access to screening tests in health care and detention settings (11). Importantly, the data have been used in ancillary studies, both alone and in combination with other national or state-level survey data. Two of these studies analyzed the impact of immigration enforcement policies on the profile, experiences, and migration plans of deported Mexican migrants (62, 63), showing that a widespread versus a prioritized enforcement strategy results in greater numbers of law-abiding individuals experiencing fear of deportation, reduced inter-state mobility, and detention and subsequently deportation as a result of minor offenses, such as traffic infractions. Other ancillary studies have shed light on health selection processes (67, 68) and educational gradients in health care access among Mexican migrants (69). The next two surveys will shed much needed light on the prevalence of, and factors associated with, NCD, mental health, and substance abuse among migrant flows traveling through the Mexico – U.S. border region. The rich dataset accumulated over more than a decade will serve to monitor changes in health and access to health services among the largest immigration population in the U.S. Data from this observatory, in combination with other population-based surveys focused on more established or non-migrant populations, will also provide clues about the effects of broad policy-level factors on health and access to services among Mexican migrants in the U.S. This work will inform future actions to improve healthcare access and reduce disparities in this population.

The Migrante Project’s research infrastructure is grounded in solid public health and social science theory frameworks that focus on both upstream macro- and micro-level health determinants and health outcomes across migration phases, at different points in time, and across international borders. This infrastructure makes it possible to monitor current health issues and respond to emerging threats, such as the ongoing COVID-19 pandemic. By having this instrument in place at the time the pandemic was reaching the Western hemisphere, we were able to collect information on COVID-19 risks, health-seeking behaviors, vaccination rates and healthcare access among migrants traveling North, South, across the border, and among those who were deported from the United States. Data on the impact of COVID-19 on migrant populations in the U.S. has been scarce and difficult to gather, but their poor living and working conditions, employment in essential labor sectors, and limited access to health services suggest they have experienced disproportionate infection rates and more severe outcomes (70). The Migrante Project was able to adapt to the circumstances of the pandemic and expand its data collection protocols to collect information on COVID-19 history, testing, prevention behaviors, and socio-economic impact. A flexible and timely data collection infrastructure can help to identify hot spots and risks for this population, healthcare and vaccine access; as well as structural determinants of health and risk for migrants.

The methodology employed by the Migrante Project is innovative in its focus on migration flows (versus migrant stocks). Furthermore, the results from the pilot longitudinal component to be tested over the next Migrante phases have the potential to inform a unique panel design for a hard-to-reach, understudied mobile population moving North and South through the Mexico-U.S. border region. The binational partnership behind the Migrante Project is critical to maximizing the potential impact of academic research on decision makers and public health praxis. Despite these strengths, the study protocol has some limitations. These include (a) the cross-sectional design, which limits the ability to establish causal inferences; (b) the expected moderate response rates, which may reduce the representativeness of the study samples; (c) the self-reported nature of most information collected in the surveys, with potential for recall and social desirability biases; (d) the implementation in only three Mexican border towns, which leaves out migrant flows crossing through other points of the Mexico-U.S. border or flying directly between the U.S. and the interior of Mexico; and (e) the relatively small sample size for specific migrant flows (e.g., Northbound migrants, Southbound migrants, etc.), which can limit statistical power for analyses involving only those flows.

The Mexico-U.S. border is a place where myriad of migratory routes converge (coming from the South beyond Mexico’s borders from other places in Central America and even other continents). Evidence suggests this region will become an even bigger migration corridor for international migrants in the 21^st^ century, especially with the U.S. economy’s increased need for foreign workers, continued violence and poverty in sending communities, and the promised shift towards more humane immigration policies by the current Biden administration. To this end, the next phases of the Migrante Project will consolidate an observatory with the potential to inform both international and local policies and programs that can respond to sudden developments (like COVID-19) and guide policies and practices to protect the health of migrants in Mexico and the U.S. in the age of migration. While the project has been expanded over time, room for growth remains. Future expansions may include extending the survey to languages other than Spanish, which would allow for inclusion of emerging migrant populations arriving to the U.S.-Mexico border, such as Haitians (71). Future surveys may also explore lowering the age limit to cover family units and unaccompanied migrant minors. The Migrante Project could also evolve to develop and test migrant health interventions to support migrants in the border region through use of mobile health applications, referral to local and remote health and social services, and linkage to protective social networks in destination communities.

Ultimately, the goal of the Migrante Project’s observatory is to be a data resource for policymakers, advocates, and scholars aiming to advance migrants’ health and increase their access to healthcare. With the continued success of this project, this methodology could become an international reference and be adapted for implementation at other busy migration corridors throughout the world.

## Data availability statement

The original contributions presented in the study are included in the article/supplementary material, further inquiries can be directed to the corresponding author.

## Ethics statement

The protocol for the Migrante Project has been reviewed and approved by the Institutional Review Board of Drexel University. All Migrante Project survey respondents provide informed consent to participate in the study.

## Author contributions

AM-D, GR, JG-F, CM-R, XZ, FL-S, CA-D, SG, and EP contributed to the conception and design of the study and helped to secure funding for this project. AM-D, CC, and LB led the writing of the manuscript. All authors were involved in the implementation and coordination of the study, reviewed drafts, and approved the final manuscript.

## Funding

Research reported in this publication was supported by the Eunice Kennedy Shriver National Institute of Child Health & Human Development of the National Institutes of Health under Award Number R01HD046886. The content is solely the responsibility of the authors and does not necessarily represent the official views of the National Institutes of Health.

## Conflict of interest

The authors declare that the research was conducted in the absence of any commercial or financial relationships that could be construed as a potential conflict of interest.

## Publisher’s note

All claims expressed in this article are solely those of the authors and do not necessarily represent those of their affiliated organizations, or those of the publisher, the editors and the reviewers. Any product that may be evaluated in this article, or claim that may be made by its manufacturer, is not guaranteed or endorsed by the publisher.

## Footnotes

^1^ www.migrante.weebly.com

^2^ www.sample-size.net

## Abbreviations

CD4+: Cluster of differentiation 4
COLEF: The College of The Northern Border (El Colegio de la Frontera Norte, COLEF, Per Its Spanish name and acronym)
COVID-19: Coronavirus disease 2019
CVD: Cardiovascular disease
Elisa: Enzyme-linked immunoassay
EMIF: Surveys on migration on The Northern and Southern Mexican Borders (Encuesta sobre Migración en las Fronteras Norte y Sur, EMIF, Per their Spanish name and acronym)
HbA1c: Hemoglobin A1c
HBsAg: Hepatitis B surface antigen
HDL: High-density lipids
Hep B: Hepatitis B
HIV: Human immunodeficiency virus
Aids: Acquired immunodeficiency syndrome
NICHD: Eunice Kennedy Shriver national institute of child health and human development
OLS: Ordinary least squares
RBC: Red blood cell
SD: Standard
SPSS: Statistical package for the social sciences
STI: Sexually transmitted infection
U.S.: United States of America
USMBHC: U.S.-Mexico border health commission
